# Melanoma mystery

**DOI:** 10.7554/eLife.22662

**Published:** 2017-01-19

**Authors:** Roger J Davis

**Affiliations:** Howard Hughes Medical Institute and the Program in Molecular Medicine, University of Massachusetts Medical School, Worcester, United Statesroger.davis@umassmed.edu

**Keywords:** Reproducibility Project: Cancer Biology, replication, metascience, reproducibility, driver mutation, melanoma, Mouse

## Abstract

Biological variability has confounded efforts to confirm the role of *PREX2* mutations in melanoma.

**Related research articles** Horrigan SK, Courville P, Sampey D, Zhou F, Cai S, Reproducibility Project: Cancer Biology. 2017. Replication Study: Melanoma genome sequencing reveals frequent *PREX2* mutations. *eLife* **6**:e21634. doi: 10.7554/eLife.21634

Chroscinski D, Sampey D, Hewitt A, Reproducibility Project: Cancer Biology. 2014. Registered Report: Melanoma genome sequencing reveals frequent *PREX2* mutations. *eLife* **3**:e04180. doi: 10.7554/eLife.04180

Melanoma is associated with DNA damage and genomic alterations caused by ultraviolet light. In 2012, as part of efforts to better understand the causes of melanoma, researchers at the Broad Institute, the Dana-Farber Cancer Institute and a number of other institutes reported the results of whole genome sequencing of 25 human metastatic melanomas (Berger et al., 2012). This analysis discovered an average of 97 structural rearrangements of the genome per tumor, and some 9,653 mutations of various types in 5,712 genes. A number of known melanoma oncogenes were identified, including *BRAF^V600E^* (in 64% of tumors) and mutated *NRAS* (36%). The analysis also found that a significant fraction of tumors contained rearrangements and mutations of a gene called *PREX2,* and experiments confirmed that cancer-associated mutations of *PREX2* promoted the growth of human melanoma cells in mice.

It has been known for a number of years that PREX2 is a GTP/GDP exchange factor that inhibits a tumor suppressor protein called PTEN, and that this process can promote tumorigenesis by activating the PI3K signaling pathway (Fine et al., 2009; Hodakoski et al., 2014; Figure 1A). More recently it has been shown that PTEN can inhibit PREX2, and that this can stop tumor cells invading tissue by preventing the activation of an enzyme called RAC (Mense et al., 2015). Moreover, cancer-associated mutations in *PREX2* disrupt these mutual inhibition processes: mutated PREX2 can still inhibit PTEN, but PTEN cannot inhibit mutated PREX2 (Mense et al., 2015; Figure 1B). All this work supports the conclusion that the over-expression of PREX2 can increase PI3K-dependent tumor growth (Fine et al., 2009), and that mutated PREX2 promotes tumorigenesis by increasing RAC-dependent invasiveness (Mense et al., 2015).

**Figure 1.**
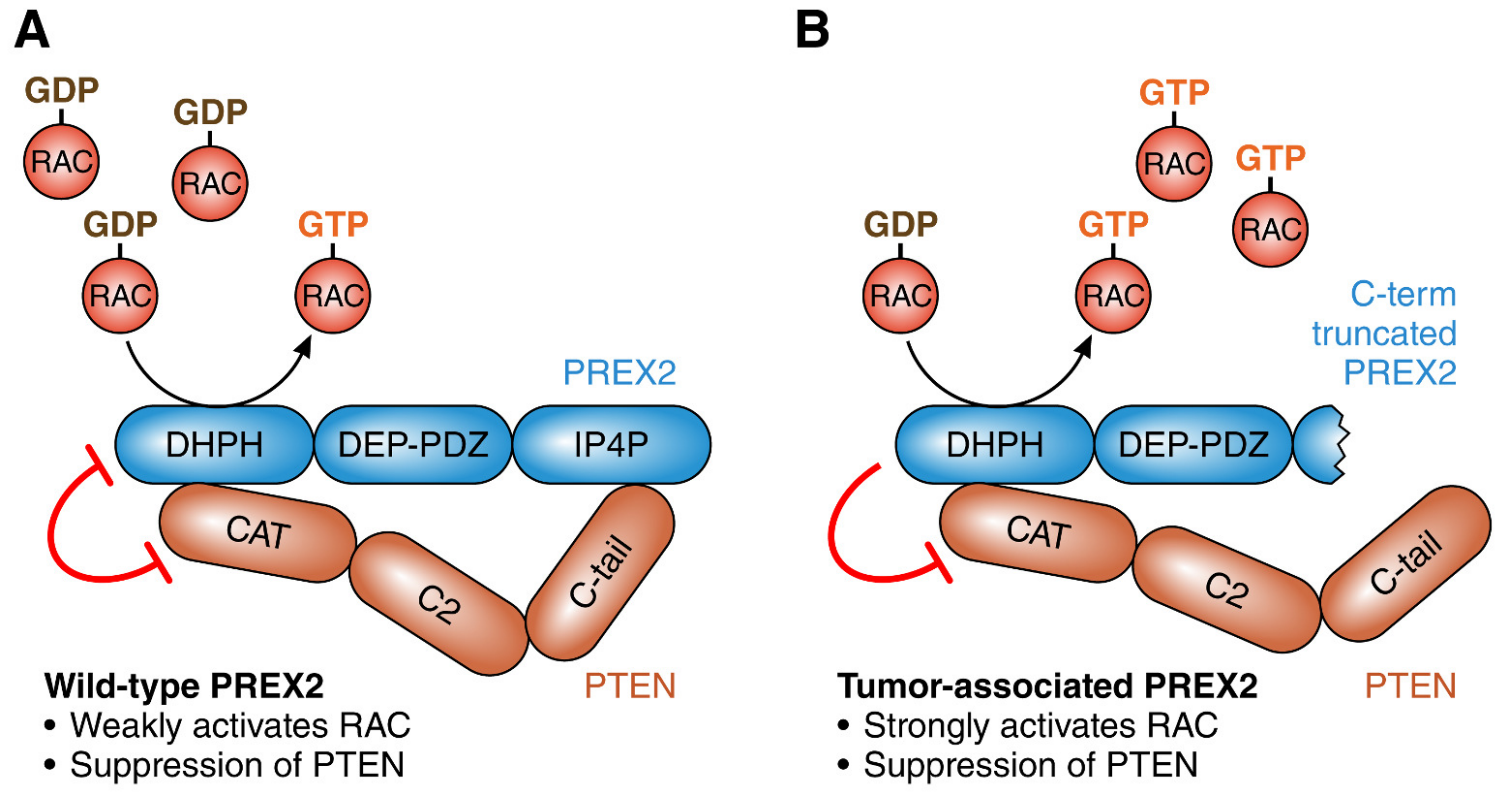
The roles of PREX2 and PTEN. (**A**) PREX2 (blue) is a GTP/GDP exchange factor that activates a GTPase called RAC. PTEN (brown) is a lipid phosphatase that suppresses tumors by inhibiting PI3K signaling (not shown). The interaction of PREX2 and PTEN (via their DHPH and CAT domains respectively) suppresses the catalytic activity of both. (**B**) Cancer-associated mutations in PREX2 (or C-terminal truncation of PREX2, as shown here) do not interfere with its ability to activate RAC or its ability to inhibit PTEN. However, PTEN is unable to inhibit mutated PREX2. Therefore mutations in PREX2 can lead to cancer by increasing both RAC and PI3K signaling. PREX2: phosphatidylinositol-3,4,5-triphosphate-dependent RAC exchange factor 2. PTEN: phosphatase and tensin homolog. RAC: RAS-related C3 botulinum toxin substrate.

As part of the Reproducibility Project: Cancer Biology, Chroscinski et al. published a Registered Report which explained in detail how they would seek to replicate selected experiments from Berger et al. (Chroscinski et al., 2014). The results of these experiments have now been published as a Replication Study (Horrigan et al., 2017).

The original paper by Berger et al. contained two major conclusions. First, *PREX2* was identified as a frequently mutated gene in human melanoma. The Reproducibility Project did not attempt to replicate this finding, but subsequent studies have reported the frequency of *PREX2* mutations in human melanoma (Hodis et al., 2012; Krauthammer et al., 2012; Marzese et al., 2014; Ni et al., 2013; Turajlic et al., 2012), including meta-analysis of 241 melanomas (Xia et al., 2014). Second, mutation of *PREX2* can accelerate human melanoma growth. The Reproducibility Project did attempt to replicate the mouse xenograft studies that support this second conclusion.

Berger et al. expressed six different mutated PREX2 proteins in TERT-immortalized human melanoma cells. These cells were transplanted into immuno-deficient mice. Control studies were performed using cells expressing either wild-type PREX2 or green fluorescent protein (GFP). Kaplan-Meier analysis demonstrated that most of the mice injected with cells expressing wild-type PREX2 or GFP exhibited tumor-free survival for more than ten weeks. In contrast, cancer-associated mutations in *PREX2* significantly reduced tumor-free mouse survival (Figures 3B and S6 of Berger et al.). The work of Berger et al. supported the conclusion that cancer-associated *PREX2* mutations can promote the growth of human melanoma cells.

Attempts to replicate these xenograft experiments were confounded by a serious technical problem. The tumors grew rapidly in the control experiments (the median time for tumor-free survival was one week) and any differences in tumor-free survival for the controls and the mice injected with cells expressing mutated PREX2 were not statistically significant (Horrigan et al., 2017). Consequently, no conclusions could be drawn concerning the possible contribution of PREX2 mutations to melanoma growth.

This Replication Study represents a cautionary tale concerning the impact of biological variability on experimental design. While strenuous efforts were made to precisely copy the experimental conditions employed in the original study, the xenografts in the Replication Study behaved in a fundamentally different way to those in the original study. The mechanistic basis for the observed differences is unclear. Presumably, there was a difference in the melanoma cells and/or the mice. Although the cells were obtained from the same source, small differences in culture conditions or passage history could have contributed to differences between the studies. Similarly, although the mice were obtained from the same source, housing the animals in a different facility may have contributed to differences between the studies.

A key lesson to be drawn from this experience is that biological variability is a critical factor in experimental design. Pilot studies to explore biological variation would have allowed this Replication Study to be redesigned to inject fewer melanoma cells and thus delay tumor growth. Biological variability means, therefore, that direct replication of a reported study might not always be the best way to assess reproducibility in certain fields.

Questions remain concerning the role of *PREX2* mutations in cancer. It is established that *PREX2* can be mutated in melanoma and pancreatic ductal adenocarcinoma (Berger et al., 2012; Waddell et al., 2015), and that cancer-associated *PREX2* mutations can promote both tumorigenesis in vivo (Lissanu Deribe et al., 2016) and tumor cell invasiveness (Mense et al., 2015). However, wild-type PREX2 can also promote tumor growth by suppressing PTEN activity and increasing PI3K signaling (Fine et al., 2009). There is a clear need for further mechanistic studies to explore the role of *PREX2* and mutations of *PREX2* in cancer.

## Note

Roger J Davis was the eLife Reviewing Editor for the Registered Report (Chroscinski et al., 2014) and the Replication Study (Horrigan et al., 2017).
